# Stepwise super minimally invasive full-thickness resection for locally advanced rectal cancer after achieving near clinical complete response with neoadjuvant therapy

**DOI:** 10.1055/a-2665-7827

**Published:** 2025-08-22

**Authors:** Yaoqian Yuan, Qianqian Chen, Shuai Tian, Kunming Lv, Jiafeng Wang, Qun Shao, Enqiang Linghu

**Affiliations:** 1 651943 Department of Gastroenterology, The First Medical Center of Chinese PLA General Hospital, Beijing, China; 2 Department of Gastroenterology, Hospital of the PLA Joint Logistic Support Force, Yantai, China; 3 104607 Department of Gastroenterology, The Second Medical Center of Chinese PLA General Hospital, Beijing, China; 4 Department of Gastroenterology, Aerospace Center Hospital, Beijing, China

Organ preservation strategies like “watch-and-wait” are increasingly used in LARC patients with cCR/near-cCR postneoadjuvant therapy 1 . Local recurrence remains critical, with 14.9–25% incidence within 2 years, necessitating effective salvage interventions 2 . While endoscopic techniques provide super minimally invasive solutions for superficial tumors 3 , the management of deep local recurrences involving or penetrating the muscularis propria presents substantial challenges. We describe an innovative stepwise super minimally invasive full-thickness resection (sft-SMIR) technique. A 75-year-old female presented with LARC in the low rectum (initial staging: cT2–3N0M0). Following short-course radiotherapy and two cycles of chemoradiotherapy, she achieved a near-clinical complete response (ncCR). After multidisciplinary discussion, sft-SMIR surgery was performed ( Fig. 1 , Video 1 ). First, the laterally spreading tumor (LST) and scars after neoadjuvant therapy were fully exposed, and the lesion area was marked ( Fig. 1 **a, b** ). Second, ESD was performed in the LST and scar surrounding areas ( Fig. 1 **c** ). Third, a traction device of a rubber band and clips was applied to fully expose the intrinsic muscle layer and deep scar areas at the lesion site. Then, the intrinsic muscle layer was incised by an electric knife, and the extracellular mesorectum was exposed ( Fig. 1 **d** ). Fourth, the intrinsic muscle layer after ESD and the full-thickness defect after EFTR were displayed ( Fig. 1 **e** ). Finally, the closure of the full-thickness defect was achieved by using clips to seal the muscle layer against the muscle layer ( Fig. 1 **f** ). The specimen was fixed and photographed with both sides ( Fig. 1 **g, h** ). Postoperative pathology suggests intramucosal carcinoma with curative resection.

**Fig. 1:**
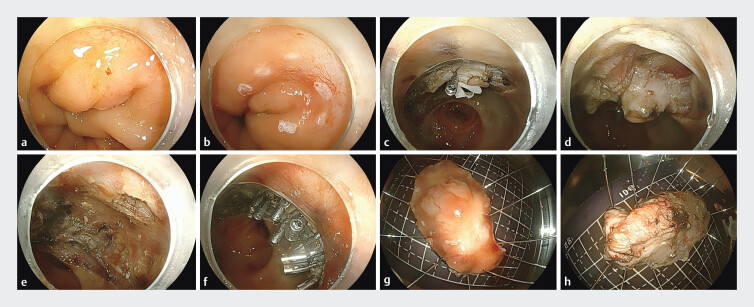
sft-SMIR for LARC after achieving ncCR with neoadjuvant therapy. The feature of LST and scars after neoadjuvant therapy was revealed under white light endoscopy. The lesion area was marked. **a** The mucosal and submucosal layers around the scar and LST were dissected. **b** The traction device was applied, and the full thickness of the rectum was resected. **c** The intrinsic muscle layer and the full-thickness defect were displayed. **d** The full-thickness defect was closed by sealing the muscularis propria with clips. **e** Application of a tissue clamp to seal the muscle layer during full-thickness excision of the wound. The mucosal layer of the gross specimen was shown. **f** The serosal layer of the gross specimen was shown. Pathological HE staining slides of lesions were shown.

Stepwise super minimally invasive full-thickness resection of LARC after neoadjuvant therapy.Video 1

The sft-SMIR technique (ESD-EFTR integration) provides a feasible, super-minimally invasive salvage strategy for patients with post-ncCR/cCR. By enabling endoscopically guided, precise full-thickness resection with controlled dissection, this approach effectively addresses deep muscularis propria involvement while substantially reducing unnecessary anal sphincter resection compared to traditional salvage surgery.

Endoscopy_UCTN_Code_CPL_1AJ_2AD_3AF

## Notes

Endoscopy E-Videos https://eref.thieme.de/e-videos
*E-Videos* is an open access online section of the journal *Endoscopy* , reporting on interesting cases and new techniques in gastroenterological endoscopy. All papers include a high-quality video and are published with a Creative Commons CC-BY license. Endoscopy E-Videos qualify for HINARI discounts and waivers and eligibility is automatically checked during the submission process. We grant 100% waivers to articles whose corresponding authors are based in Group A countries and 50% waivers to those who are based in Group B countries as classified by Research4Life (see: https://www.research4life.org/access/eligibility/ ). This section has its own submission website at https://mc.manuscriptcentral.com/e-videos .
